# The NR2F1-Related 5q14.3–q21.1 deletion causing periventricular heterotopia with cerebral visual impairment: a longitudinal case report and genotype–phenotype analysis

**DOI:** 10.3389/fgene.2026.1793726

**Published:** 2026-05-07

**Authors:** Helen St Clair Tracy, Gordon N. Dutton, Hildegard Nikki Hall, Andrew Blaikie

**Affiliations:** 1 Infection and Global Health Division, School of Medicine, University of St Andrews, St Andrews, United Kingdom; 2 Glasgow Caledonian University, Glasgow, United Kingdom; 3 Ophthalmology Department, Queen Margaret Hospital, NHS Fife, Kirkcaldy, United Kingdom

**Keywords:** Balint syndrome, cerebral visual impairment, genotype–phenotype correlation, longitudinal case study, neurodevelopmental disorder, NR2F1 haploinsufficiency, optic nerve abnormalities, periventricular heterotopia

## Abstract

Large interstitial deletions spanning chromosome 5q14.3 to q21.1 and encompassing *NR2F1* are rare. Existing descriptions have largely focused on structural features and presenting manifestations, with fewer reports examining functional neurodevelopmental outcomes over time, particularly in individuals with large interstitial deletions. Here, we present a 16-year longitudinal analysis of an individual with a *de novo* 13.58 Mb interstitial deletion of 5q14.3 to q21.1 encompassing *NR2F1* (ClinVar accession SCV007328941), consistent with NR2F1-related neurodevelopmental disorder, historically described under OMIM:615722 (Bosch-Boonstra-Schaaf optic atrophy syndrome). We integrate longitudinal clinical, visual, neurological, and developmental data to examine relationships between optic nerve findings, periventricular heterotopia (PH; OMIM:612881, chromosome 5q14.3 deletion syndrome), cerebral visual impairment (CVI), epilepsy, hypotonia, and long-term functional outcomes. The index case manifested PH and severe CVI from infancy, with profound hypotonia associated oromotor and airway dysfunction. Epilepsy first manifested during adolescence. Longitudinal in-depth analysis suggests that CVI may represent a key mediating factor underlying cognitive, behavioral, and communicative difficulties. Substantial latent cognitive capacity was revealed once visual complexity was reduced and environments appropriately adapted. Analysis of published cases indicates that PH is an uncommon but recurrent feature of *NR2F1* haploinsufficiency and suggests that optic atrophy can be secondary to cerebral visual pathway dysfunction. This case highlights that longitudinal functional assessment can enhance genotype–phenotype interpretation in rare genomic disorders and provide clinically actionable insights for diagnosis, management, and outcome prediction.

## 1 Introduction

Chromosome 5q14.3 contains *NR2F1*, a dosage-sensitive gene essential for cortical development, neuronal migration, and visual pathway organization. Haploinsufficiency of *NR2F1* causes NR2F1-NDD, a condition associated with variable combinations of CVI, optic nerve abnormalities, epilepsy, neurodevelopmental delay, and behavioral features (Schaaf et al., 2022). Early descriptions emphasized optic atrophy; however, increasing evidence supports a broader and more heterogeneous neurodevelopmental phenotype.

Reported neuroimaging findings in NR2F1-NDD include corpus callosum thinning, white matter abnormalities, and, less frequently, PH (Bertacchi et al., 2022). Visual impairment is common, yet its functional basis is often poorly characterized. Although optic disc pallor or hypoplasia is frequently reported, CVI is increasingly recognized as a core component of the phenotype, including in individuals with preserved or only mildly reduced visual acuity (Jurkute et al., 2021). CVI may therefore be under-recognized, particularly when visual acuity appears typical (Chandna et al., 2021). Epilepsy and apparent intellectual disability are also common features (Schaaf et al., 2022), further complicating phenotypic interpretation.

Large interstitial deletions spanning 5q14.3 to q21.1 and encompassing *NR2F1* are rare. At the time of writing, 11 individuals with deletions of this size and genomic interval are recorded in the DECIPHER database (Foreman et al., 2023), and most published reports are cross-sectional. Consequently, little is known about longitudinal functional trajectories or how early developmental presentations, particularly visual impairment, may be misinterpreted over time.

Here, we report a male individual (T1) with a *de novo* 13.58 Mb interstitial deletion of 5q14.3–q21.1 encompassing *NR2F1*, presenting a detailed 16-year longitudinal clinical analysis. Integrated visual, neurological, developmental, and functional data are used to refine interpretation of CVI, PH, optic nerve findings, and epilepsy. This report presents evidence consistent with the hypothesis that, within NR2F1-NDD, CVI may function as a key mediating factor underlying cognitive, communicative, and behavioral outcomes rather than as a secondary or coincidental feature. By integrating functional observation with genomic interpretation, we aim to clarify how CVI may influence phenotype expression and clinical trajectory in this disorder.

## 2 Patient information

The proband (T1) has a heterozygous *de novo* interstitial deletion of chromosome 5q14.3–q21.1 spanning chr5:89,411,425–103,021,765 (NCBI Build 36, GRCh38/hg18), encompassing *NR2F1* among 38 deleted genes (Supplementary Material S1). Initial G-banded cytogenetic analysis suggested a broader interstitial deletion of chromosome 5q14.2–q21.1, which was subsequently refined by array comparative genomic hybridization (array-CGH). The variant has since been harmonized to GRCh38 coordinates (chr5:90,079,852–103,658,165) and is recorded in DECIPHER as a pathogenic *de novo* deletion encompassing neighboring genes. The copy number variant has been deposited in ClinVar under accession SCV007328941. No additional pathogenic sequence variants were identified.

He was born at term following a pregnancy notable only for abnormal first-trimester maternal serum screening; chorionic villus sampling excluded common aneuploidies. There was no reported family history of intellectual disability, epilepsy, neurodevelopmental disorder, or structural brain abnormalities.

## 3 Clinical findings

Clinical examination in infancy and early childhood revealed profound hypotonia and marked global developmental delay. Early clinical concerns included absent visual fixation and feeding difficulties, and visual impairment was identified in early infancy. Feeding difficulties were associated with poor oromotor control and airway instability. Flexible nasendoscopy demonstrated pharyngomalacia with partial upper airway obstruction, alongside laryngomalacia and mild tracheomalacia, consistent with generalized hypotonia.

Repeated ophthalmologic examinations through early childhood described structurally normal anterior segments and optic discs, with no evidence of primary ocular pathology, despite severe functional visual impairment.

**FIGURE 1:**
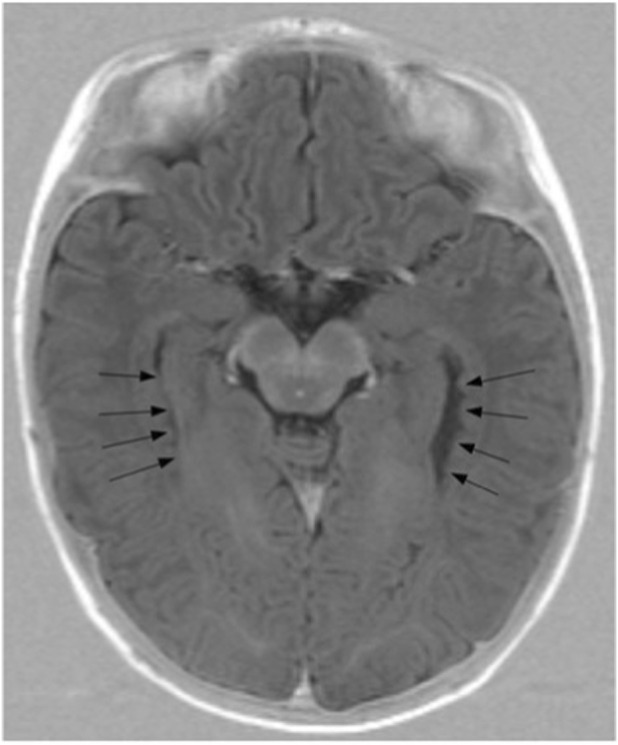
Axial MRI of T1 at the level of the temporal horns demonstrating multiple nodules of heterotopic grey matter lining the lateral walls of the temporal horns of the lateral ventricles bilaterally (black arrows).

Neurologic examination was limited by hypotonia, sensory dysregulation, and poor tolerance of handling. Early paroxysmal behaviors prompted concern for epilepsy; however, serial electroencephalography did not demonstrate epileptiform activity. Neuroimaging later identified PH (Figure 1), while epileptic seizures emerged only in adolescence as focal-onset seizures with secondary generalization.

Clinical, developmental, ophthalmologic, and neurologic findings were documented longitudinally through routine and specialist care. A chronological overview is provided in the Timeline (Table 1), with full clinical detail presented in Supplementary Material S2, S3.

## 4 Timeline

**TABLE 1:** Chronological summary of key clinical, developmental, neuroimaging, and neurological findings in T1 from the antenatal period to 16 years of age. Ages are approximate where indicated. Detailed clinical descriptions are provided in Supplementary Material S1, S2.

Age	Key findings
Antenatal	Abnormal first-trimester maternal serum screening indicating increased risk for trisomies 13 and 18; chorionic villus sampling excluded common aneuploidies
Birth–6 weeks	Term delivery at 38 weeks. Early hypotonia, feeding difficulties, airway malacia, persistent distress, and poor state regulation. Absent visual fixation noted
3 months	Ophthalmological and electrophysiological findings consistent with severe visual impairment; registered as severely sight impaired (blind) on the basis of cortical blindness. Cerebral visual impairment (CVI) was not formally identified or labelled at this stage
4–5 months	Array comparative genomic hybridization identified a *de novo* interstitial deletion spanning 5q14.3–q21.1. Because of proximity to the adenomatous polyposis coli *APC* gene, additional targeted testing was undertaken to define deletion breakpoints and exclude *APC* involvement
5–6 months	MRI demonstrated bilateral periventricular nodular heterotopia and qualitatively slender optic nerves and chiasm
6–12 months	Electrophysiology demonstrated normal electroretinograms with profoundly abnormal or absent visual evoked potentials, supporting a post-retinal cause of visual impairment
Early childhood	Visual impairment was regarded as secondary to global developmental delay rather than as a primary contributor to functional difficulties
3 years	Independent walking achieved in the context of hypotonia, joint hypermobility, and visual field deficits. Persistent feeding difficulties continued
Mid-childhood (∼5 years)	Caregivers became aware of cerebral visual impairment as a diagnostic framework, understanding the visual difficulties as brain-based; however, severity and specific functional implications were not yet fully characterized
7 years	Comprehensive neuro-ophthalmological assessment identified severe cerebral visual impairment with profound dorsal stream dysfunction, consistent with Balint syndrome. This assessment directly informed targeted environmental and educational adaptations
9 years	Neuro-ophthalmologic follow-up demonstrated gradual improvement in visual acuity and functional adaptation in the context of cerebral visual impairment, with persistent lower visual field loss, reduced contrast sensitivity, and Balint syndrome
Adolescence (∼13 years)	Onset of focal-onset seizures evolving to bilateral tonic–clonic epilepsy
16 years (current)	Epilepsy improved but not fully controlled, with approximately three seizures per year on treatment. Severe cerebral visual impairment and multisensory impairment persist, with meaningful functional gains in engagement, communication, and emotional regulation following CVI-informed adaptations

## 5 Diagnostic assessment

Profound early neurodevelopmental impairment limited assessment and contributed to diagnostic uncertainty. Early visual impairment was labelled as cortical blindness without functional characterization, and visual difficulties were regarded for several years as secondary to global developmental delay. In this case, delayed recognition of CVI as a potential key mediating factor in learning and behavior was associated with substantial downstream functional consequences.

Paroxysmal behaviors were initially interpreted to be epileptic despite normal electroencephalography. Neuroimaging described qualitatively slender optic nerves, while repeated ophthalmological examinations demonstrated normal optic discs and retinae, creating ambiguity regarding structural versus post retinal contributions to visual dysfunction.

Repeated early invasive assessments contributed to persistent aversion to clinical environments. The absence of developmentally and psychologically informed approaches delayed recognition of severe CVI, particularly dorsal stream dysfunction in its most severe form (Balint syndrome). Full clinical detail is provided in Supplementary Material S2, S3.

## 6 Therapeutic interventions

Therapeutic interventions evolved over time in response to emerging clinical understanding and are summarized below in chronological sequence.

Early management was supportive and symptomatic, focusing on feeding support, airway management, and global developmental therapy. Persistent feeding difficulties required gastrostomy placement for nutrition, hydration, and medication administration. Developmental input targeted gross motor skills, postural control, and functional mobility within a general neurodevelopmental framework.

During early childhood, visual impairment was interpreted as secondary to global developmental disability. Management of visual impairment initially occurred within standard ophthalmological follow-up, without CVI-specific environmental or educational adaptations.

From mid-childhood, increasing distress and difficulty with transitions prompted consideration of autism spectrum disorder; these behaviors were later considered to be consistent with severe CVI, communication barriers, and environmental mismatch. Following recognition and detailed characterization of CVI, including Balint syndrome, therapeutic focus shifted toward reducing visual complexity, supporting visual attention, and prioritizing spoken language as the primary mode of communication.

Antiseizure medications were intermittently used in early childhood because of concern for epilepsy. Sodium valproate was discontinued due to sedation and absence of electrographic evidence. With the emergence of definitive focal-onset seizures in adolescence, lamotrigine was introduced but provided limited seizure control. As seizure semiology became clearer, carbamazepine was commenced and was associated with a substantial reduction in seizure frequency. Lamotrigine was subsequently phased out, and carbamazepine remains the primary antiseizure medication, with dose adjustments guided by clinical response. Alongside antiseizure management, multidisciplinary educational and therapeutic supports addressed motor delay, communication, and sensory processing differences. No disease-modifying therapies were available.

Overall, management shifted from supportive symptomatic care in infancy to targeted CVI-informed environmental adaptation and epilepsy management in adolescence. Changes in intervention were guided by evolving diagnostic clarification and longitudinal functional response. Full intervention details are provided in Supplementary Material S2.

## 7 Follow-up and outcomes

Longitudinal follow-up occurred through routine clinical care, specialist review where tolerated, and ongoing educational support. Outcomes were assessed through clinician evaluation (including neurological and repeat neuro-ophthalmologic assessment), caregiver report, educational documentation, and observed functional response to intervention across home and school settings.

Following recognition of CVI and implementation of CVI-informed environmental and educational adaptations, T1 demonstrated an immediate and sustained positive response. Reduced visual clutter, highly structured routines, and presentation of one element at a time were associated with improved engagement, behavioral regulation, and tolerance of interaction. These adaptations were implemented consistently across home and educational settings, with sustained caregiver and educational adherence.

Over time, these adaptations supported further functional gains. By adolescence, T1 demonstrated substantial receptive language development, with comprehension of hundreds of spoken words and phrases. Expressive verbal language remains limited, though a small number of spoken words are used. Despite severe CVI, T1 developed selective functional use of visually mediated and tactile technologies under tightly controlled conditions, including structured television menus, use of a computer keyboard by touch, and limited iPad use; these skills were confined to highly structured visual contexts and did not extend to visually complex environments.

Behavioral regulation improved substantially, with physical or reactive behaviors now rare and typically associated only with periods of heightened stress or sensory overload. These improvements were temporally associated with enhanced visual and communicative access rather than resolution of underlying neurodevelopmental impairment.

Severe CVI and auditory processing difficulties persist. Repeat neuro-ophthalmologic follow-up confirms ongoing visual field loss, reduced visual acuity and contrast sensitivity, and profound dorsal stream dysfunction consistent with Balint syndrome. Epilepsy, which emerged in adolescence, has improved with pharmacologic treatment but remains incompletely controlled. Antiepileptic treatment has been tolerated without significant adverse effects. Tolerance of clinical environments remains limited, and medical care continues to require trauma-informed planning.

Overall, longitudinal follow-up suggests that while the behavioral and developmental effects of early diagnostic delay could not be reversed, recognition of CVI and provision of structured, low-complexity environments were associated with meaningful access to learning, communication, and social understanding despite persistent severe neurodevelopmental disability.

This report has several strengths and limitations. The principal strength lies in the longitudinal depth of clinical observation, integration of genomic data with functional assessment, and detailed characterization of visual and developmental trajectory. However, as a single-case report, causal inferences cannot be established, and genotype–phenotype relationships remain hypothesis-generating. Neuroimaging interpretation was qualitative, and formal quantitative longitudinal measures were not consistently feasible due to clinical tolerability. Findings therefore require confirmation in larger cohorts.

## 8 Discussion

This longitudinal case provides an opportunity to examine how a large 5q14.3–q21.1 deletion encompassing *NR2F1* unfolds clinically over time. While multiple neurological and structural features are present, including epilepsy, hypotonia, and periventricular heterotopia, the functional trajectory suggests that CVI may represent a potential key mediator of cognitive, communicative, and behavioral outcome within NR2F1-NDD.

The following sections consider individual phenotypic domains within this broader interpretive framework.

### 8.1 Genotype–phenotype correlation and deletion size

Across reported individuals with deletions ≥10 Mb, including a 17 Mb deletion (Cardoso et al., 2009), a ∼13 Mb deletion (Sobreira et al., 2009), and the present 13.58 Mb deletion in T1, global developmental delay and epilepsy were recurrent features, while structural brain abnormalities were variably present. In contrast, smaller deletions encompassing *NR2F1*, including those under 1 Mb, demonstrate a comparable core phenotype. Al-Kateb et al. described a 582 kb deletion including *NR2F1* associated with bilateral optic atrophy, visual perceptual impairment, and global developmental delay (Al-Kateb et al., 2013). Similarly, Chen et al. reported three individuals with deletions ranging from 0.2 to 0.9 Mb, all exhibiting developmental delay and optic nerve atrophy, with hypotonia present in two of three cases (Chen et al., 2016). Furthermore, fifteen individuals with heterozygous pathogenic *NR2F1* sequence variants reported by Chen et al. showed consistent developmental delay with frequent optic nerve abnormalities, while hypotonia and epilepsy were variably observed (Chen et al., 2016). Taken together, the phenotypic overlap observed across large deletions, microdeletions, and single-gene variants suggests that the core neurodevelopmental and visual features do not scale proportionally with deletion size and supports a central role for *NR2F1* haploinsufficiency, although modifying contributions from additional deleted genes cannot be excluded.

To directly address whether additional genes within the deleted interval may contribute to phenotype expression, we systematically reviewed all 38 protein-coding genes encompassed by the 5q14.3–q21.1 deletion (Supplementary Material Table S1). Gene–disease validity, inheritance pattern, and dosage sensitivity were assessed using ClinGen and OMIM.

Among all deleted genes, *NR2F1* is the only gene with an established monoallelic disease association and recognized haploinsufficiency as a pathogenic mechanism relevant to heterozygous deletion. Pathogenic variants in *NR2F1* cause an autosomal dominant neurodevelopmental disorder historically described as Bosch-Boonstra-Schaaf optic atrophy syndrome (OMIM:615722). The broader phenotype of NR2F1-NDD extends beyond optic atrophy and includes CVI, developmental delay, hypotonia, epilepsy and corpus callosum abnormalities, features all present in T1.

In contrast, several other deleted genes, including *ARSK*, *KIAA0825*, *PPIP5K2*, *TTC37* (also known as *SKIC3*), and *PCSK1*, are associated with autosomal recessive disorders requiring biallelic pathogenic variants. The phenotypes described in these recessive conditions are not consistent with T1’s clinical presentation and would not be expected to manifest in the context of a heterozygous deletion.

*CHD1*, which has an autosomal dominant disease association, causes Pilarowski-Bjornsson syndrome (OMIM:617682), characterized primarily by developmental delay, speech apraxia, seizures, and variable autistic features (Pilarowski et al., 2018). Current evidence suggests that pathogenic *CHD1* variants may act through dominant-negative or missense mechanisms rather than through haploinsufficiency (Pilarowski et al., 2018). ClinGen currently assigns limited evidence for *CHD1* haploinsufficiency, and CVI is not a defining feature of *CHD1*-related disease.

The majority of remaining genes within the interval have no established monogenic disease association or lack evidence for dosage sensitivity.

Taken together, current gene–disease evidence supports *NR2F1* haploinsufficiency as the most parsimonious primary driver of T1’s phenotype, while acknowledging that subtle modifying contributions from additional deleted genes cannot be definitively excluded.

### 8.2 Periventricular heterotopia

Within the broader framework of CVI as a mediator of functional outcome in NR2F1-NDD, structural cortical abnormalities such as PH warrant consideration in relation to visual pathway development. To our knowledge, CVI has not previously been explicitly reported in individuals with PH within this genomic region.

Periventricular heterotopia (PH) is a malformation of cortical development characterized by ectopic nodules of grey matter lining the lateral ventricles due to disrupted neuronal migration. The condition is genetically heterogeneous, with pathogenic variants in *FLNA* and *ARFGEF2* genes (Cardoso et al., 2009).

Cardoso et al. defined a 5.8 Mb critical region and proposed several positional candidate genes, including *GPR98* (also known as *ADGRV1*), *CETN3*, *MCTP1*, and *NR2F1* (Cardoso et al., 2009). At the time, limited gene–disease data were available. Since then, *ADGRV1* has been established as a cause of autosomal recessive disease, without evidence for a monoallelic haploinsufficiency mechanism. In contrast, *NR2F1* is now recognized as a dosage-sensitive gene with a confirmed autosomal dominant neurodevelopmental disorder associated with heterozygous loss of function. All individuals reported with PH within the 5q14.3 region, including T1, have had heterozygous deletions. While contiguous gene effects cannot be excluded, current gene–disease evidence strengthens the candidacy of *NR2F1* as a biologically plausible contributor to PH within this interval.

In T1, PH was identified in the context of a larger interstitial deletion spanning 5q14.3 to q21.1 that overlaps the critical region described by Cardoso et al. All reported individuals with PH within this region, including T1, have deletions encompassing *NR2F1*. *NR2F1* encodes a nuclear receptor essential for cortical development, neuronal differentiation, and axonal guidance, and is highly expressed in the developing cerebral cortex, optic nerve, and thalamus (Bosch et al., 2014).

Cortical malformations have been reported in individuals with pathogenic *NR2F1* variants, including medial temporal and perisylvian dysgyria, corpus callosum thinning, and white matter abnormalities, although PH was not observed in that series (Desai et al., 2023), possibly reflecting phenotypic variability or under recognition. Experimental data support a migration-related mechanism, as *Nr2f1* haploinsufficiency in a mouse model resulted in downregulation of *Netrin1*, a key axon guidance cue, with downstream misrouting of retinal ganglion cell axons (Jurkute et al., 2021), a process that could plausibly disrupt cortical neuronal migration consistent with the presence of PH in T1.

While *NR2F1* remains a strong candidate gene within this interval, the restriction of reported PH to larger deletions, and its absence in smaller deletions or sequence variants involving *NR2F1* alone, suggests caution in attributing PH solely to *NR2F1* haploinsufficiency. The possibility of a contiguous gene effect warrants consideration.

### 8.3 Epilepsy

Within the broader neurodevelopmental phenotype associated with NR2F1-NDD deletions, epilepsy is a recognized feature, with considerable variability in age of onset, seizure type, and severity. It has been reported in individuals with periventricular heterotopia (Cardoso et al., 2009) as well as in those with *NR2F1* haploinsufficiency without documented cortical malformations (Chen et al., 2016; Rech et al., 2020; Engels et al., 2009), indicating that seizure susceptibility is likely related to *NR2F1* haploinsufficiency or broader cortical disruption rather than to PH alone.

In T1, epilepsy emerged in adolescence and presented as focal seizures with secondary generalization. Seizure frequency improved with carbamazepine. Earlier paroxysmal events in infancy and early childhood, initially suspected to be epileptic, were subsequently reinterpreted as non-epileptic behaviors occurring in the context of severe, unrecognized cerebral visual impairment (see Supplementary Material Table S2).

Bertacchi et al., in their review, discuss the possibility that severe epileptic infantile spasms could contribute to neurodevelopmental impairments, including visual dysfunction (Bertacchi et al., 2022), but this assertion has yet to be supported by longitudinal data concerning possible onset of CVI following epilepsy in NR2F1-related disorder. Epilepsy and CVI frequently co-occur (Jimenez Gómez et al., 2022; Demarest et al., 2019); however, available evidence is largely cross-sectional and has yet to be established as a temporal sequel.

In T1, severe CVI from birth was documented. Epilepsy only emerged in adolescence. In this case the CVI is considered a primary brain-based visual impairment in association with epilepsy of later onset.

Taken together, epilepsy appears to be a recurrent but variably expressed feature within the spectrum of NR2F1-related neurodevelopmental disorder. However, its presence should be interpreted alongside, rather than used to explain, associated cortical visual dysfunction.

### 8.4 Vision: optic nerve abnormalities, cerebral visual impairment and iris coloboma

Visual findings in NR2F1-NDD may include both optic nerve abnormalities and CVI (Desai et al., 2023; Jurkute et al., 2021). In many reported cases, optic nerve changes such as pallor or hypoplasia are taken as primary explanatory findings (Jurkute et al., 2021). T1 represents an informative example in which severe CVI was present in the context of largely normal ophthalmologic examination, alongside ambiguous neuroimaging findings. This provides an opportunity to examine the relationship between structural and functional visual findings and to highlight the need for careful interpretation of optic nerve abnormalities in this condition.

Desai et al. reported that 50% (10 of 20) of individuals with *NR2F1*-related neurodevelopmental disorder (NR2F1-NDD) had a clinical diagnosis of CVI, while 86% showed optic atrophy or optic disc pallor (Desai et al., 2023). These findings support an association between *NR2F1* haploinsufficiency and structural visual system anomalies but also highlight that CVI may be underrecognized, particularly when visual acuity appears typical (Chandna et al., 2021). This interpretation is consistent with findings from Jurkute et al., who reported preserved or only mildly reduced visual acuity in most of their 22 cases despite clear evidence of visual brain abnormality (Jurkute et al., 2021). Notably, 84% of the Jurkute cohort met diagnostic criteria for autism spectrum disorder, a condition associated with CVI (Chokron et al., 2020). CVI has also been independently linked to intellectual and developmental delay (Black et al., 2019; Williams et al., 2021; Jayasinghe et al., 2025), both of which were present in 84% of the Desai cohort. Together, these findings suggest that CVI may be underrecognized in NR2F1-NDD, particularly in the absence of ocular signs or when behavioral features overlap with autism or broader neurodevelopmental disability. The present case, T1, with severe CVI, contributes to this evolving understanding. Recent systematic review and network analyses have also identified *NR2F1* among genes implicated in neurodevelopmental disorders with ophthalmologic manifestations (Shinsato et al., 2024).

Bosch et al. proposed that optic nerve atrophy and hypoplasia in NR2F1-NDD may, in some individuals, result from retrograde trans synaptic degeneration secondary to cerebral visual pathway dysfunction (Bosch et al., 2014). Jacobson et al. provided supporting evidence, demonstrating ganglion cell layer thinning consistent with retrograde trans synaptic degeneration in children with CVI, regardless of whether the underlying cerebral insult was congenital or acquired (Jacobson et al., 2019). These findings indicate that optic nerve and retinal changes in NR2F1-NDD may reflect both congenital miswiring and secondary degeneration arising from disrupted cerebral visual development.

Optic atrophy is often assumed to be secondary in conditions associated with intra uterine brain developmental abnormalities (Mudgil and Repka, 2000; Hellström, 1999), and it is plausible that other forms of atypical brain development, including periventricular heterotopia (PH), could similarly contribute to optic nerve abnormalities. However, evidence also supports a primary developmental contribution to optic nerve pathology in NR2F1-NDD. Jurkute et al. demonstrated that *NR2F1* is essential for retinal ganglion cell differentiation and optic nerve axon guidance (Jurkute et al., 2021). In a *Nr2f1* ^+/−^ mouse model, haploinsufficiency disrupted molecular guidance cues such as Netrin 1, resulting in retinal ganglion cell misrouting, impaired migration, and optic nerve hypoplasia. Together with observations of retinal ganglion cell thinning and optic nerve hypoplasia in human subjects, these findings link *NR2F1* dosage directly to optic nerve development and indicate that both primary developmental mechanisms and secondary processes, including retrograde trans synaptic degeneration, remain plausible.

The clinical trajectory in T1 illustrates the interpretive complexity of visual pathway findings in NR2F1-NDD. Ophthalmologic examinations from 6 weeks to 5 years of age consistently described healthy optic discs and retinae, with no evidence of primary ocular pathology. By contrast, MRI at 5 months reported qualitatively slender optic nerves and optic chiasm, with a recommendation for correlation with visual function. As T1 was already registered as severely sight impaired at that time, this contextual knowledge may have influenced radiological interpretation. The qualitative nature of the MRI description, including the phrasing “appear slender,” underscores its subjectivity. These findings therefore require caution, as repeated ophthalmologic examinations demonstrated normal ocular structure, while severe functional visual impairment was attributable to CVI rather than ocular disease.

Collectively, available evidence supports a model in which cerebral visual pathway dysfunction may precede and contribute to optic nerve and retinal changes in individuals with deletions spanning 5q14.3 to q21.1 or other forms of *NR2F1* haploinsufficiency. In T1, it remains uncertain whether optic nerve changes have occurred and, if so, whether they are primary, secondary, related to retrograde trans synaptic degeneration, a combination of these mechanisms, or absent.

To illustrate this complexity, a published individual with a 5q14.3 to q15 deletion reported by Engels et al. retained both copies of *NR2F1* yet exhibited bilateral optic atrophy together with cerebellar vermis aplasia, corpus callosum abnormalities, choroid plexus cysts, and occipital horn dilatation. This indicates that optic nerve involvement can occur within the 5q14.3 region in the absence of *NR2F1* haploinsufficiency and may arise through broader cortical or structural brain mechanisms. These observations support a cautious interpretation of optic nerve findings in 5q14.3 deletions and highlight the need to consider additional genes and developmental pathways rather than attributing visual pathway anomalies solely to *NR2F1*.

In summary, optic nerve abnormalities in NR2F1-NDD may arise through multiple mechanisms, including primary developmental disruption related to *NR2F1* haploinsufficiency, secondary effects associated with broader structural brain abnormalities, and specific mechanisms such as retrograde trans-synaptic degeneration.

Iris coloboma has been reported in individuals with overlapping 5q14.3 to q15 deletions (Cardoso et al., 2009; Sobreira et al., 2009) and in an individual with a pathogenic *NR2F1* sequence variant (Gazdagh et al., 2022). Chorioretinal coloboma has been reported in one individual with a pathogenic *NR2F1* sequence variant (Gazdagh et al., 2022); however, chorioretinal or optic nerve colobomata have not been described to date in individuals with larger 5q14.3 to q21.1 deletions involving *NR2F1*.

Together, these findings indicate a broader and more mechanistically complex spectrum of visual and ocular involvement in 5q14.3 deletion disorders than previously recognized. They support an integrated model in which cerebral visual pathway dysfunction, optic nerve development, and broader cortical malformations interact within a shared developmental framework associated with *NR2F1* haploinsufficiency and the surrounding genomic region.

### 8.5 Clinical features: oromotor function and breathing

Beyond visual dysfunction, additional systemic features also influenced early functional trajectory in T1, particularly hypotonia and its effects on oromotor and airway function. Hypotonia is a consistent feature of 5q14.3–q21.1 deletions, including those involving *NR2F1* (Cardoso et al., 2009; Kaiwar et al., 2017; Desai et al., 2023). In T1, profound infantile hypotonia was associated with persistent feeding difficulties and clinically significant airway involvement. Flexible nasendoscopy demonstrated pharyngomalacia with partial upper airway obstruction, alongside laryngomalacia and elements of tracheomalacia. These findings are most coherently explained by generalized low muscle tone resulting in dynamic airway collapse rather than fixed structural abnormality.

Similar pharyngeal muscle weakness and feeding difficulties have been reported in other individuals with overlapping deletions (Engels et al., 2009; Desai et al., 2023), with hypersalivation also described. In hypotonia-associated neurodevelopmental conditions, hypersalivation could reflect impaired oropharyngeal clearance and inefficient swallowing rather than increased saliva production, a mechanism also recognized in disorders affecting motor control of swallowing, including Parkinson’s disease (Nascimento et al., 2025). Comparable hypotonia-related airway mechanisms are recognized in other neurodevelopmental disorders, such as Prader–Willi syndrome, where laryngomalacia and pharyngomalacia are established sequelae of low muscle tone (Com et al., 2023).

Taken together, these findings suggest that hypotonia-related oromotor and airway dysfunction may be under-recognized contributors to feeding and respiratory difficulties in NR2F1-NDD. Recognition of this mechanism has direct clinical relevance, supporting early consideration of dynamic airway assessment and swallow function evaluation rather than attributing symptoms to non-specific feeding intolerance alone.

### 8.6 Developmental phenotype

The developmental phenotype in NR2F1-NDD is often characterized as fixed intellectual disability; however, longitudinal reassessment in T1 suggests a more dynamic interpretation when severe CVI is recognized and accommodated.

Developmental delay and intellectual disability have been widely reported in individuals with 5q14.3 to q21.1 deletions (Bosch et al., 2014; Kaiwar et al., 2017). In T1, retrospective longitudinal review indicates that early concerns, including absence of speech, apparent seizure-like behaviors, emotional dysregulation, anxiety, and profound learning difficulties, were later understood to arise largely in the context of severe CVI rather than from an independent or fixed intellectual disability (Supplementary Material Table S2).

At 6 years of age, the family became aware of Balint syndrome, a severe manifestation of dorsal stream dysfunction within the CVI spectrum (Gillen and Dutton, 2003). This condition affects visuospatial attention, visually guided movement, and visual scene integration, rendering complex or cluttered environments inaccessible (Goodale and Milner, 1992; Lueck and Dutton, 2015; St Clair Tracy et al., 2025). Introduction of structured, low-complexity environments using a one-thing-at-a-time approach, initially within a low-stimulation single-color enclosure (Little and Dutton, 2015), together with slow, consistent spoken language, resulted in a marked change in functional engagement. Whilst the association between CVI-informed adaptations and functional change was striking, as this report describes a single individual, causality cannot be definitively established. The longitudinal depth strengthens inference, but these findings should be interpreted as hypothesis-generating and require confirmation in larger cohorts.

Before these adaptations, T1 functioned developmentally at a level comparable to a six-month-old infant. Following accommodation of visual processing limitations, a striking cognitive strength emerged in the form of exceptional memory, which had previously been inaccessible. Desai et al. similarly reported unusually strong memory abilities in 82% of individuals with *NR2F1* related neurodevelopmental disorder (Desai et al., 2023), suggesting that such skills may be systematically overlooked in the context of complex neurodevelopmental disability.

## 9 Conclusion

This case provides longitudinal observations consistent with the hypothesis that CVI may represent a key mediating mechanism underlying developmental, behavioral, and communicative difficulties in NR2F1-NDD rather than a coincidental or secondary feature. When unrecognized, CVI may profoundly constrain learning and adaptive progress. When identified, it may provide a clear, actionable target for tailored environmental, educational, and therapeutic intervention, even in the presence of severe neurodevelopmental disability.

This case illustrates the value of longitudinal clinical observation in NR2F1-RDD, demonstrating how key features may emerge, evolve, or be misinterpreted over time and therefore be missed in cross-sectional assessment. In T1, epilepsy emerged only in adolescence, while the effects of severe functional visual impairment were initially misattributed to global developmental delay, highlighting the importance of developmental timing in phenotypic interpretation.

Integration of longitudinal medical data with observations from families, educational settings, and therapeutic contexts can substantially refine phenotypic interpretation in NR2F1-NDD, particularly for features with delayed or context-dependent expression such as CVI.

For clinicians and researchers using genetic case reports to inform assessment and counselling, these findings underscore the importance of avoiding early attribution of functional limitations to fixed intellectual disability or epilepsy in NR2F1-NDD. Careful consideration of CVI as a mediating mechanism may materially alter diagnostic interpretation, prognostic counselling, and support planning.

## Data Availability

The datasets presented in this study are publicly available. This data can be found in ClinVar with the accession number SCV007328941, at https://www.ncbi.nlm.nih.gov/clinvar/variation/4685561/?oq=SCV007328941&m=GRCh38%2Fhg38+5q14.3-21.2(chr5:90079852-103658165)x1.

## Supplementary material

SUPPLEMENTARY MATERIAL S1 Table of all protein-coding genes within the GRCh38-defined 5q14.3–q21.1 deletion interval in T1, including gene–disease validity, inheritance pattern, and classification of relevance to phenotype.

SUPPLEMENTARY MATERIAL S2 Detailed clinical and developmental case report, including antenatal history, genetic findings, ophthalmological and neurological features, developmental profile, and longitudinal clinical observations.

SUPPLEMENTARY MATERIAL S3 Neuro-ophthalmological assessment reports at ages 7 and 9, detailing visual acuity, visual fields, contrast sensitivity, visual perception, and functional implications for learning and behaviour.

SUPPLEMENTARY MATERIAL S4 CARE checklist outlining adherence to case report reporting standards, including clinical information, diagnostic assessment, intervent, and outcomes. It has been pointed out that genome build nomenclature should use the formal GRCh terminology for consistency.
