# Supplementary material for: The NR2F1-Related 5q14.3–q21.1 deletion causing periventricular heterotopia with cerebral visual impairment: a longitudinal case report and genotype–phenotype analysis
Source: Front Genet. 2026 May 7;17:1793726. doi: 10.3389/fgene.2026.1793726 (PMC13189929; doi:10.3389/fgene.2026.1793726)
Supplement: Supplementary file 1 [file DataSheet2.pdf]

Supplementary Information 1 – Detailed Clinical and Developmental Case Report

1. **Antenatal History**
2. **Birth and Neonatal Period**
3. **Emerging Concerns and Initial Visual Observations**
4. **Genetic Findings**
5. **Ophthalmology**
6. **Neurology and Epilepsy**
7. **Otorhinolaryngology (ENT) and Upper Airway**
8. **Additional Clinical Findings**
9. **Development**

Case Report

This case describes a 16 year old male individual with a heterozygous interstitial deletion spanning 5q14.3 to 5q21.1. This deletion encompasses 38 genes, including several with functions yet to be characterised, as well as genes implicated in neurodevelopment and tumour suppression.

To our knowledge, this precise genetic profile has not hitherto been reported. The proband presents with a combination of features both consistent with, and divergent from, those reported in seven comparable cases identified in the literature. This report provides a detailed clinical and developmental history, organised by system and domain, to facilitate phenotypic comparison and expand understanding of the spectrum of expression associated with this deletion.

The following sections describe the proband in detail, covering genetic findings, ophthalmological and neurological features (including epilepsy), medical and physical characteristics, developmental profile, and other relevant observations.

1. Antenatal History

The proband was conceived to a 40-year-old mother. The pregnancy was unremarkable apart from maternal nausea and migraine. First trimester combined screening indicated a low risk for trisomy 21 based on nuchal translucency measurement. However, maternal serum biochemistry (PAPP-A and β-hCG) indicated an increased risk for trisomies 13 and 18, with an estimated risk of 1 in 40.

Chorionic villus sampling returned a normal result, excluding common aneuploidies, including trisomy 13 (Patau syndrome) and trisomy 18 (Edwards syndrome).

2. Birth and Neonatal Period

The proband was delivered at 38 weeks' gestation via planned induction. The pregnancy was classified as high risk due to a maternal history of premature rupture of membranes in previous pregnancies. This was the mother's fourth pregnancy. The proband's three siblings have no known genetic disorders.

Birthweight was 3.63 kg (8 lbs), placing him within the upper centiles for gestational age. Immediately after delivery, respiratory compromise was observed but resolved spontaneously without resuscitation.

During the first week of life, the proband lost 0.25 kg and failed to regain birthweight within the expected timeframe. Despite prior maternal breastfeeding experience, feeding was difficult, with poor latch and frequent dribbling. Over the first six weeks of life, the infant demonstrated persistent distress, with intense crying episodes, difficulty settling, poor sleep and noisy breathing prompting multiple presentations to primary care services. Initial symptoms were attributed to colic or gastro-oesophageal reflux. Subsequent assessment identified laryngomalacia, pharyngomalacia, and mild tracheomalacia.

3. Emerging Concerns and Initial Visual Observations

As the infant grew, further clinical concerns emerged. Unlike his siblings, he exhibited persistent distress, alternating between prolonged crying and exhaustion, with minimal time spent in a calm, alert state. He was difficult to soothe and unable to sleep independently.

Due to the severity of his early presentation, visual concerns were not initially apparent. Concerns regarding visual responsiveness arose when a private paediatrician, consulted in the context of ongoing respiratory difficulties, noted an absence of visual tracking.

A referral to the local hospital's rapid-access clinic had previously been initiated but had not been actioned due to a clerical processing error. With respiratory symptoms worsening, the family sought private medical assessment. The paediatrician conducted an airway ultrasound to assess safety for home discharge and formally documented absent visual tracking.

At six weeks of age, the infant was reviewed by a private ophthalmologist who suspected delayed visual maturation and recommended reassessment after six weeks. At an urgent general practitioner review the infant was admitted to hospital for further investigation. During this period, oxygen saturation was recorded at 72%. Macrocephaly with a bulging fontanelle was also noted. Flexible nasendoscopy identified pharyngomalacia contributing to partial airway obstruction. Feeding was deemed unsafe, and a nasogastric (NG) tube was inserted. Genetic testing results became available during admission, prompting referral to clinical genetics and additional specialist services.

4. Genetic Findings

Array comparative genomic hybridisation (array-CGH) was carried out owing to the above concerns. The array confirmed a de novo heterozygous interstitial deletion of the long arm of chromosome 5, spanning 5q14.3 to 5q21.1, approximately 13.6 Mb in size (chr5:89,411,425–103,021,765; NCBI Build 36, hg18).

Initial G-banded cytogenetic analysis had suggested a broader interstitial deletion of chromosome 5q14.2–q21.2. Subsequent array-CGH refined the breakpoints to 5q14.3–q21.1. The variant has since been harmonised to GRCh38 coordinates (chr5:90,079,852–103,658,165) and is recorded in DECIPHER as a pathogenic de novo deletion encompassing *NR2F1* and neighbouring genes.

The analysis was performed using the Agilent 44K oligonucleotide array (designed by the National Genetics Reference Laboratory, Wessex) and confirmed a cytogenetically visible deletion. Quality metrics (DRS score 0.183834) were within the 'excellent 'range.

Neither parent carried the deletion, confirming it to be *de novo*.

The deletion does not include the APC gene, which was initially queried. However, it does encompass 37 known genes, including two associated with entries in the OMIM morbid gene catalogue:

• ADGRV1 (formerly GPR98/VLGR1) – associated with Usher syndrome type IIC and linked to sensory processing
• PCSK1 – associated with endocrine and metabolic disorders, including obesity and endocrine dysfunction
Other notable genes within the deleted interval include:
• NR2F1 – associated with Bosch–Boonstra–Schaaf optic atrophy syndrome, potentially relevant to the observed visual phenotype
• TTC37 – implicated in trichohepatoenteric syndrome, though phenotypic overlap in this case is limited
• CHD1 – encodes a chromatin remodelling factor frequently deleted in prostate cancer; although its pathogenic relevance in the germline context remains uncertain, its presence in this deletion is of interest
• CAST, ERAP1/2, LIX1 – with proposed or emerging roles in neurodevelopment, immune regulation, and intracellular signalling

Deletions involving this region have previously been associated with hypotonia and neurodevelopmental delay (Schinzel, 2001), features also present in this individual.
The case has been submitted to the DECIPHER database (ID: WSX251190).

5. Ophthalmology

At six weeks of age, ophthalmological examination revealed absent fixation, poor pupillary reactivity, and mild bilateral hypermetropia. The anterior segments were healthy, and fundoscopy confirmed normal optic discs and retinae. Based on these findings, delayed visual maturation was initially suspected, and orthoptic follow-up arranged.

At five months, electrodiagnostic testing was performed to evaluate functional visual pathway integrity. Flash ERGs, although partially obscured by artefact, showed no evidence of generalised retinal dysfunction. These findings suggested significant post-retinal dysfunction, prompting further testing. Binocular VEP flash stimulation failed to elicit consistent responses. Monocular flash VEPs were variable, with a delayed positive component identified at approximately 270 ms (right eye, 12 μV) and 280 ms (left eye, 8 μV).Normal ranges: Latency: 100-150msec. Amplitude: 20-50mv. These findings indicated markedly delayed conduction and reduced signal strength, consistent with significant post-retinal dysfunction.

By six months, the infant remained unable to fix or follow targets beyond light sources, although there was some light response with slow incomplete pupil reactions. The fundi continued to appear normal, and he was formally registered as severely sight impaired. Repeat electrophysiology at 12 months demonstrated absence of flash VEPs bilaterally, while ERG results were normal. These results confirmed cortical blindness (cerebral visual impairment (CVI)), consistent with post-retinal visual pathway dysfunction.
At 21 months, further clinical assessment identified intermittent exotropia and nystagmus. Visual behaviour remained markedly limited, with only light tracking evident. No response to more complex visual stimuli was observed.

The retinae and optic nerves were repeatedly described as structurally normal on direct ophthalmological examination from six weeks to five years of age, with good visualisation of the optic discs by multiple clinicians. However, an MRI performed at five months reported that the optic nerves and chiasm "appear slender," and suggested correlation with visual function. As the child had already been registered as severely sight impaired at that time, the radiologist was aware of this diagnosis, raising the possibility that the interpretation was influenced by clinical expectation. Notably, the MRI findings were qualitative rather than quantitatively measured, and the use of subjective terminology such as "appear" limits their diagnostic weight. The apparent discrepancy between MRI and fundoscopy findings may reflect the inherent difficulty in detecting subtle or symmetric bilateral optic nerve thinning, particularly in infants, or it may indicate that no structural abnormality was present. This case highlights the challenge of distinguishing true optic nerve pathology from interpretive bias or artefact, especially when clinical assessments and imaging findings diverge.

Follow-up Ophthalmological Evaluations (Ages 7 and 9)
At age 7, detailed neuro-ophthalmological evaluation confirmed profound visual impairment consistent with CVI. Binocular visual acuity was estimated at approximately 6/200, with a marked reduction in contrast sensitivity (46%). T1's visual field was restricted to the upper field; no responses were noted in the lower field unless stimuli were brought into the horizontal midline. He mis-reached in the upper visual field and collided with low-lying objects, indicating impaired visual guidance of movement. He responded preferentially to high-contrast, slowly moving targets and was observed to attend to only one or two visual elements at a time, features consistent with Balint syndrome, the most severe form of dorsal stream dysfunction. Despite these limitations, he demonstrated strong visual memory, enabling him to learn routes and navigate familiar spaces with increasing confidence.

By age 9, visual acuity had improved to an estimated 6/75. This gradual improvement in function, accompanied by the development of compensatory strategies (such as positioning himself below visual targets to optimise upper visual field use), is consistent with Stage 2 Delayed Visual Maturation (DVM) within the underlying diagnosis of CVI. Progress in visual awareness, planning, and functional engagement with visual environments, including digital media, were evident. However, while visual function had improved considerably through adaptation, key impairments remained unchanged. Contrast sensitivity remained markedly reduced, the lower visual field impairment was as severe as before, and features consistent with Balint syndrome persisted. Improvements were therefore primarily attributable to functional adaptation rather than neurological recovery.

Full reports from these evaluations, detailing methodology and educational recommendations, are included in Supplementary Information 2.

6. Neurology and Epilepsy

6.a Neuroimaging findings

MRI performed under general anaesthetic using a standard protocol for children under two years revealed age-appropriate myelination. The optic nerves and chiasm were noted to be slender, raising the possibility of structural contributions to the observed visual impairment. (However, given the electrophysiological evidence of post-retinal dysfunction and the clinical absence of optic nerve atrophy, these findings are interpreted as secondary or contributory, rather than primary causes of visual deficit). Bilateral multiple nodules of heterotopic grey matter lining the lateral walls of the temporal horns of the lateral ventricles, were evident (Figure 1). These findings are consistent with periventricular nodular heterotopia (PH), a neuronal migration disorder during development, in which grey matter fails to reach the cortical plate. The frontal horns of the lateral ventricles appeared more prominent than the occipital horns, although this was considered a non-specific feature. The septum pellucidum was present, while the corpus callosum appeared somewhat thinned posteriorly.

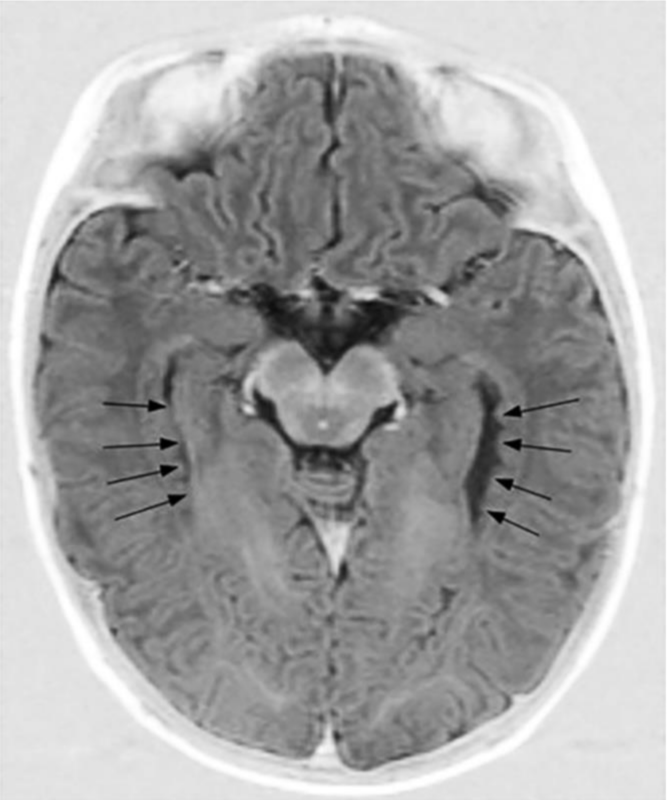

Legend] Image 1: Axial MRI at the level of the temporal horns demonstrating multiple nodules of heterotopic grey matter lining the lateral walls of the temporal horns bilaterally (black arrows).

6.b Epilepsy

At 20 months of age, owing to a series of jerking episodes and periods of unresponsiveness, the proband was reviewed by a paediatric neurologist, who concluded that the clinical presentation was consistent with early-onset symptomatic epilepsy. The events included limb jerks (sometimes involving the abdomen), prolonged tonic eye deviation, unresponsiveness, and screaming spells without clear triggers. While some episodes were associated with febrile illness, others were afebrile and were nocturnal. PH provides a potential anatomical focus for seizure activity.

Although initial EEG was normal, the recurrence of episodes prompted a trial of anticonvulsant medication. Sodium valproate was initiated at a dose of 20 mg/kg/day, titrated from 60 mg to 120 mg twice daily. During the consultation, the neurologist discussed the elevated risk of sudden unexpected death in epilepsy (SUDEP) associated with uncontrolled symptomatic epilepsy. While the estimated SUDEP risk in the general paediatric epilepsy population is approximately 1 in 10,000, this risk rises to 1 in 400–500 in individuals with refractory symptomatic epilepsy. The family agreed to proceed with treatment, and follow-up was arranged.

Over the following months, the episodes persisted despite medication, and concerns arose concerning the child's increased lethargy and reduced responsiveness. 24-hour video telemetry EEG was performed at two years of age, again showing no evidence of epileptiform activity. Several of the child's typical behaviours—including eye movements, stiffening, and vocalisations—were observed during the study, but none was associated with any electrographic change. The EEG was again interpreted as normal, with age-appropriate background and sleep architecture. In light of these findings, the sodium valproate was withdrawn, and the behaviours were later reinterpreted as manifestations of CVI rather than being epileptic in origin.

6.c Adolescent-Onset Epilepsy

At the age of 13 years, the proband developed focal-onset seizures evolving to bilateral tonic–clonic activity, with two events occurring approximately one month apart, each lasting between one and four minutes. These episodes marked a clear departure from the earlier paroxysmal events of infancy, which had been non-epileptic in origin. The seizures emerged in the context of a known chromosomal 5q deletion and the underlying structural brain anomaly, specifically periventricular nodular heterotopia. Although EEG was considered, it could not be tolerated and was therefore not performed. A clinical diagnosis of epilepsy was made based on seizure semiology and its recurrence.

Initial treatment comprised lamotrigine, titrated to 200 mg twice daily (approximately 6 mg/kg/day). Despite this, seizure frequency remained high, typically occurring every three to four weeks, with occasional clustering. The clinical pattern was consistent with focal-onset seizures with secondary generalisation. Events were consistently preceded by a profound pre-ictal "disconnect" phase, characterised by sudden unresponsiveness, complete apnoea, and cyanosis within seconds. This phase involved immediate collapse, often with falls, and could last up to two minutes. It was followed by a bilateral tonic–clonic seizure lasting up to five minutes. The severity of this autonomic disruption was thought to play a role in seizure generation and informed subsequent adjustment in antiepileptic therapy.

Carbamazepine was introduced, targeting both focal cortical excitability and potential autonomic involvement. Following this change in treatment, seizure frequency reduced markedly to two or three episodes per year. A magnesium supplement was also introduced, and caregivers noted a subsequent reduction in the duration and severity of post-ictal recovery periods.

7. Otorhinolaryngology (ENT) and Upper Airway

7.a. Audiology and Hearing Function

The proband passed newborn hearing screening aged one month. However, soon after, concerns emerged regarding both under-responsiveness, and episodes of apparent hyperacusis, including panic-like behaviours in reaction to everyday sounds. Objective testing throughout early childhood produced varied results, complicated by comorbid hypotonia and profound visual impairment.

At 2 years and 4 months, bilateral grommets were inserted (alongside percutaneous endoscopic gastrostomy (PEG) tube placement). Following this procedure, hearing responses improved, indicating increased auditory input. However, a marked escalation in distress behaviours was observed, including sudden outbursts and episodes of agitation. These behaviours were temporally associated with environmental sound exposure and were later interpreted within the broader context of CVI.

Bone conduction hearing aid trials were initiated because of persistent bilateral middle ear effusions and an inconsistent behavioural hearing profile. An auditory brainstem response (ABR) under general anaesthetic demonstrated hearing thresholds in the satisfactory range (1–4 kHz bilaterally), confirming intact cochlear function and middle ear mechanisms.

Despite satisfactory auditory thresholds on ABR testing, the proband continued to exhibit extreme inconsistent responses to sound. These reactions included distress and agitation in response to common environmental noises, but at other times, no observable responses to loud auditory stimuli were evident. At three years of age, following formal assessment by multisensory impairment (MSI) consultants, congenital multisensory impairment, also termed deafblindness, was identified, based on significant functional impairment in both hearing and vision despite structurally intact pathways.

The variability in auditory responsiveness was subsequently re-interpreted as evidence of atypical auditory processing, likely involving disrupted integration within the parietal cortex, where auditory stimuli are spatially mapped, attended to, and accorded meaning. These findings were understood in the context of the proband's CVI, with overlapping cerebral involvement contributing to his broader multisensory integration difficulties. Cerebral auditory impairment was deemed the likely explanation.

7.b ENT and Feeding

Feeding difficulties were evident from early infancy. Breastfeeding was unsuccessful, and by six weeks of age, formula feeds were introduced due to poor weight gain. Oral intake remained limited, with frequent spillage, poor coordination, and occasional episodes of choking. At three months, during admission for a lower respiratory tract infection, a nasogastric (NG) tube was inserted in response to faltering weight, which had dropped below the 9th percentile. NG feeding was poorly tolerated, with repeated tube dislodgement. The proband had a pre-existing diagnosis of pharyngomalacia, which may have contributed to reduced airway tolerance of the NG tube.

Owing to persistent intolerance of nasogastric feeding, oral intake was trialled using thickened formula based on carob bean gum (Carobel), which was better tolerated. Feeding remained slow, but weight stabilised. At approximately one year of age, a high-calorie prescription formula (Nutrini) was introduced via bottle. Despite adequate nutritional intake, oral feeding beyond the bottle remained limited. Small quantities of puréed foods or yoghurt were occasionally accepted, but significant oral aversion persisted, and the proband did not exhibit typical food-seeking behaviours.

Oral administration of medication was problematic due to significant oral aversion. Sodium valproate, prescribed for suspected epilepsy, and other essential medications could not be reliably delivered orally. During episodes of feeding refusal or intercurrent illness, hospital admissions were required for intravenous fluids and medication support. At 2 years

and 3 months, a percutaneous endoscopic gastrostomy (PEG) was performed to provide reliable access for medication administration and hydration. Following PEG placement, the frequency of hospital admissions decreased. By age three, clinical assessment confirmed immature oral motor function, with feeding difficulties attributed to sensory and behavioural factors rather than structural dysphagia.

Videofluoroscopy was not performed, as aspiration risk was considered low. While under general anaesthetic for gastrostomy placement, endoscopy confirmed gastro-oesophageal reflux (GOR), which was subsequently managed with long-term proton pump inhibitor therapy (Omeprazole). During the same admission, blood tests revealed low iron levels despite the use of a nutritionally complete prescription formula (Nutrini). Iron supplementation was initiated and required on an ongoing basis, consistent with impaired iron absorption or metabolism.

7.c. Sleep and Sleep–Wake Regulation

Disrupted sleep has been a persistent clinical feature. From infancy through early childhood, the proband exhibited a markedly disorganised sleep–wake pattern, with daily episodes of prolonged night-waking lasting two to three hours. Pharyngomalacia, stridor, and apnoeic episodes were present in early life. A formal sleep study at age two excluded sleep-disordered breathing but identified occasional short central apnoeic episodes (maximum duration: 10.6 seconds), considered developmentally typical and not requiring intervention.

Despite the absence of sleep-disordered breathing, sleep difficulties remained severe and prolonged. Recurrent night-waking persisted beyond early childhood. These difficulties were later interpreted within the context of CVI.

8. Additional Clinical Findings

8.a. Cardiovascular

Transthoracic echocardiography at four months of age demonstrated structurally normal cardiac anatomy and function. A small left-to-right atrial shunt was noted, raising the possibility of a patent foramen ovale (PFO). This was asymptomatic and has not required follow-up.

8.b Renal

Renal tract ultrasound was performed in infancy to investigate a suspected anomaly; no abnormalities were identified.

8.c. Bowel Function and Constipation

Chronic constipation has been a persistent feature since infancy. Bowel movements were prolonged and associated with straining and seizure-like posturing, though these episodes were not associated with epileptiform activity on EEG. Management with polyethylene glycol (Movicol) was initiated in early childhood and remains ongoing.

8.d. Musculoskeletal/Spinal Findings

Spine radiographs identified six non-rib-bearing lumbar vertebrae. This is considered a normal anatomical variant; however, vertebral anomalies have been described in individuals with deletions involving this region.

8.e. Hypotonia and Connective Tissue Findings

Physiotherapy assessment at approximately one year of age confirmed profound hypotonia with a proximal-to-distal gradient. Truncal and cervical tone were markedly reduced, with poor co-contraction of stabilising muscle groups. Tolerance of anti-gravity positions was limited, and selective motor control was immature. Passive joint range of motion was full, with generalised hypermobility observed across major limb joints. These features were considered contributory to delayed motor development and reduced postural stability.

Independent walking was achieved at three years of age. Hypotonia and joint hypermobility remain prominent. Ligamentous laxity is particularly marked at the elbows and knees, with hyperextension observed in multiple planes.

8.f. Dermatology
The proband exhibits chronic skin sensitivity with a tendency toward ulceration and delayed healing. When present, skin lesions are persistent and prone to secondary irritation. Episodes of skin deterioration are typically localised but can be prolonged without treatment.

8.g. Physical Features
The proband demonstrates several dysmorphic features, some of which overlap with those reported in individuals with interstitial deletions involving 5q14.3–q21.1:

- High, broad forehead
- Frontal bossing, most prominent in infancy
- Hypertelorism (widely spaced eyes)
- Mild downward slant of the palpebral fissures
- Broad nasal bridge
- Small, low-set ears
- Misaligned upper central incisors with abnormal angulation and midline divergence; tooth morphology otherwise normal
- Long, tapered fingers
- Second toe longer than the hallux (Morton's toe)
- Small feet (UK adult shoe size 3.5 at 16 years), below the expected range for age and sex
- Short stature (height 161 cm at 16 years, below the 2nd centile)

9. **Development**

9.a Growth Parameters
Weight gain was initially compromised due to early feeding difficulties but stabilised following nutritional interventions in infancy. Birthweight was 3.63 kg (upper centiles for gestational age). The following weight trajectory was recorded:

**Age**	Birth	5 days	2 weeks	4 weeks	8 weeks	9 weeks	12 weeks
**Weight (kg)**	3.63	3.38	3.4	3.45	4.2	4.61	5.02

This pattern reflects early faltering growth, followed by gradual stabilisation over the first three months of life.

Since that time, weight has tracked consistently around the 50th percentile. Head circumference reached the 98th percentile within the first year of life, leading to a clinical diagnosis of macrocephaly, which has persisted.

9.b.Motor Development
Gross motor milestones were significantly delayed. Independent walking was achieved at three years of age, though gait remains unsteady with poor postural control.

CVI with reduced visual acuity contributed to motor difficulties. A lower visual field deficit with associated neglect affected the entire inferior field, impacting awareness of foot placement and ground surface. Left-sided visual inattention was present, with frequent collisions on that side. Impaired visually guided movement (optic ataxia) further affected both fine and gross motor function. Use of mobility aids such as a cane has not been established.

Neuromuscular factors compound motor challenges. Marked hypotonia, particularly involving the trunk and neck, combined with macrocephaly, contributed to delayed postural control and motor fatigue. A persistent preference for lying on the floor was noted in early childhood.
Fine motor development has been similarly affected by the combined influence of CVI and hypotonia. Despite these limitations, the proband demonstrates functional use of technology for communication and interaction, utilising a combination of tactile strategies and spatial memory. Fine and gross motor accuracy remain limited in unfamiliar tasks or environments.

9.c. Learning and Cognition
At six years of age, the proband was non-verbal and demonstrated global developmental delay, functioning at a level comparable to early infancy. Play was limited to simple exploratory behaviours consistent with toys designed for infants aged six months or younger. Profound cerebral visual and auditory processing impairments were identified as significant barriers to engagement and learning. Formal recognition and support of these multimodal impairments through environmental adaptations and consistent language use were associated with subsequent cognitive gains.

Educational strategies included reducing visual complexity, enhancing auditory access, and introducing consistent, simplified language. The proband demonstrated improved engagement with these adaptations. As a young adult, he now speaks a small number of words, demonstrates comprehension of additional vocabulary, and reliably follows simple verbal instructions. He recognises a limited set of written words and displays sustained interest in basic number recognition.
Despite significant learning disability and multisensory impairment, cognitive strengths have emerged, particularly in memory domains. Visual, verbal, and tactile memory support information acquisition when presented within a structured, consistent framework.

9.d. Independence and Daily Living Skills
The proband requires support across all areas of personal care. By age 16, he achieved partial independence with feeding, including use of a spoon and cup with assistance. He also drinks independently from a bottle and manages certain handheld foods. Despite early oral aversion, functional feeding skills have improved.

He remains doubly incontinent with minimal awareness of toileting needs. Continence care requires full adult assistance, though the proband participates by adjusting position when prompted. Cooperation is facilitated by comprehension of simple verbal instructions.

All aspects of personal hygiene require supervision and support. He is able to stand unaided in the shower but cannot independently perform hygiene tasks. He follows basic instructions during care routines and is generally cooperative. Toothbrushing is poorly tolerated, attributed to longstanding facial sensitivity and tactile defensiveness. Oral health remains good, supported by a sugar-free diet.
Although functionally dependent for most daily tasks, participation is enhanced by structured routines and familiar prompts.

9.e. Behavioural Profile
Sensory Processing and Emotional Regulation
The proband demonstrates a complex sensory profile consistent with multisensory impairment. Longstanding tactile sensitivity, particularly involving the face, is present. Auditory over-responsiveness has been observed, with distress in response to everyday environmental sounds. Visual overload, disorientation in unfamiliar environments, and reduced

tolerance for unpredictability contribute to anxiety-like behaviours. Improvements in emotional regulation have been associated with environmental adaptations, including strategies supporting CVI and communication.

Challenging Behaviour and Pharmacological Management

During a period of environmental change, including family relocation and school transition, the proband exhibited sustained behavioural challenges characterised by scratching, biting, and hair-pulling. These behaviours were most pronounced around transitions, particularly school attendance. Changing school environments mitigated some triggers; however, behavioural difficulties persisted. Pharmacological management included sertraline to support emotional regulation and chloral hydrate for sleep. Sertraline was associated with reduced distress behaviours but was gradually tapered under psychiatric supervision and discontinued by age eight. Chloral hydrate was discontinued due to limited benefit and concerns regarding dependency.

Social Understanding and Personality

Improvements in language comprehension have been accompanied by increased emotional regulation and social engagement. The proband demonstrates basic social reciprocity, seeks shared interactions, and displays humour-oriented behaviours. Behavioural outbursts occasionally occur under high-stress conditions or when the proband is unable to communicate distress, consistent with sensory processing and communication limitations.

9.f. Communication

Communication has been a significant developmental challenge for the proband. At one year of age, he was non-verbal but demonstrated emerging receptive language, indicated by appropriate responses to familiar auditory cues, such as the family dog’s name. Early communication interventions involved multi-modal systems, including tactile cues, songs, signs, and objects of reference, but these approaches were not effective.

By six years of age, he remained non-verbal and did not demonstrate consistent responses to structured communication systems. Multisensory processing limitations, including reduced ability to integrate simultaneous sensory input, were identified as a primary barrier to communication. A simplified, sequential communication approach, minimising competing sensory demands, was subsequently introduced.

Following this adaptation, the proband acquired a limited spoken vocabulary and demonstrates understanding of simple verbal instructions, particularly within the context of personal care and daily routines. He is able to recognise a small set of written words but does not generate written output. This structured approach to communication supports emotional regulation, learning, and safety awareness. Alternative communication systems were discontinued due to limited efficacy in the context of his sensory processing profile.
