# Supplementary material for: The NR2F1-Related 5q14.3–q21.1 deletion causing periventricular heterotopia with cerebral visual impairment: a longitudinal case report and genotype–phenotype analysis
Source: Front Genet. 2026 May 7;17:1793726. doi: 10.3389/fgene.2026.1793726 (PMC13189929; doi:10.3389/fgene.2026.1793726)
Supplement: Supplementary file 2 [file DataSheet4.pdf]

CARE Checklist

Title:
The diagnosis and primary genomic finding are stated in the title, which identifies the manuscript as a case report and specifies the NR2F1-related 5q14.3–q21.1 deletion.

Keywords:
Key diagnoses and features are listed in the Keywords section, including NR2F1 haploinsufficiency, cerebral visual impairment, periventricular heterotopia, and longitudinal case report.

Abstract:
The Abstract summarises what is unique about this case, namely a 16-year longitudinal functional analysis of a rare interstitial 5q14.3–q21.1 deletion encompassing *NR2F1*. It outlines the patient's main clinical features, diagnostic findings, longitudinal outcomes, and the key clinical lessons arising from the case.

Introduction:
The Introduction explains the background to NR2F1-related neurodevelopmental disorder, outlines existing gaps in the literature, and clarifies why this longitudinal case provides novel and clinically relevant insight.

Patient Information:
De-identified patient information, including genetic findings, early developmental history, and relevant medical background, is presented in the Patient Information section. Family history and psychosocial context are described where relevant.

Clinical Findings:
Significant physical, neurological, ophthalmological, and developmental findings are described in the Clinical Findings section, including hypotonia, feeding and airway involvement, and severe cerebral visual impairment.

Timeline:
Historical and current clinical information is organised in a chronological timeline (Table 1), summarising key developmental, diagnostic, and clinical milestones.

Diagnostic Assessment:
Diagnostic methods, including genetic testing, neuroimaging, electrophysiology, and ophthalmological assessment, are described in the Diagnostic Assessment section. Diagnostic challenges and differential interpretations over time are discussed.

Therapeutic Interventions:
Therapeutic and supportive interventions, including feeding support, airway management, developmental therapies, educational adaptations, and antiepileptic treatment, are described in the Therapeutic Interventions section.

Follow-up and Outcomes:
Longitudinal follow-up and outcomes are described in the Follow-up and Outcomes section, including functional changes associated with recognition of cerebral visual impairment and implementation of CVI-informed adaptations.

Discussion:
The Discussion provides a scientific analysis of the case in the context of existing literature, addressing genotype–phenotype relationships, periventricular heterotopia, epilepsy, visual system involvement, and

developmental outcomes. Strengths and limitations of the case report are considered, and the rationale for the conclusions is explained.

Patient Perspective:
Not included. Due to the individual's severe communication impairment, a direct patient perspective could not be meaningfully obtained. The case is therefore presented using longitudinal caregiver observation and clinical documentation.

Informed Consent:
Written informed consent for publication of clinical information and images was obtained from the participant's legal guardian, as stated in the Consent to Publish section.
