# Supplementary material for: The NR2F1-Related 5q14.3–q21.1 deletion causing periventricular heterotopia with cerebral visual impairment: a longitudinal case report and genotype–phenotype analysis
Source: Front Genet. 2026 May 7;17:1793726. doi: 10.3389/fgene.2026.1793726 (PMC13189929; doi:10.3389/fgene.2026.1793726)
Supplement: Supplementary file 3 [file DataSheet3.pdf]

Age 7

Information about how T1 currently sees and what this means

Key findings

T1's clarity of vision (visual acuity) and ability to differentiate shades of grey (contrast sensitivity)
The thickness of high contrast lines and gaps between lines that T1 can just see at a distance of 25cm is 2.5mm.
If he is to see at this level up to 50cm the line thickness and minimum gaps between lines that is visible to T1 is 5.0mm.
Moreover, T1 responded only to the Cardiff card with the highest level of contrast sensitivity.

The area over which T1 can see
T1 reacts to moving objects in his upper visual field, but not his lower visual field.
This is corroborated by the fact that while not colliding with objects extending into his upper visual field, he tripped over objects on the floor.

T1's perception of movement
While watching a TV screen from very close up, T1 detects slowly moving targets that instantly attract his visual attention. However, he does not respond to quickly moving targets that typical children of T1's age would.

T1's perception of colour
T1 is described as being attracted towards and bright primary colours, particularly yellow and orange.

T1's recognition of what he can sees
T1 is described as not recognising close family members by means of vision, but being able to know them through the sounds of their voices.

T1's navigation
T1 is described as being able to progressively build up his knowledge of routes, so that he now knows his way around both his home and school.

T1's ability to use his vision to guide his movement
T1 mis-reaches for objects when reaching out for them in his upper visual fields.

T1's ability to be aware of more than one or two items at once
There are a number of pieces of evidence that T1 may be able to give attention to only one or two items at once. These are:

- His lack of reaction to moving targets within his lower visual field,
- His inaccuracy of visual guidance of movement and
- His tendency to collide with objects when his attention is elsewhere

…are associated in the majority of cases with such vision.
His behaviour while watching TV is to get very close, and to shift his attention from one item to another. This too is typical of the visual behaviour of children who are aware of only one or two items at once at any one time.

T1's visual memory
As already mentioned, T1 is able to find his way around from memory.
This indicates that although T1's vision is lower than that of other people, he is able to progressively build up his visual memories in a way that is useful to him.

Helping T1 to make best use of his vision

T1 knows his vision to be his normal. He has not lost any vision, but his visual function is significantly limited.
Vision is used for three main purposes, guiding movement of the limbs and body through the environment, accessing information, whether it is nearby or in the distance, and interacting socially.

Guidance of movement
T1's low visual acuity, and low contrast sensitivity mean that low contrast obstacles (such as the living room table which is the same colour brown as the floor) may not be seen even when T1 is looking at it, unless there are contrasting white or light coloured objects on it.

His inability to see below the midline unless he tips his head down or looks down or both, mean that floors need to be clear of obstacles and low furniture is best not moved, unless T1 is involved in the exercise himself.

His impaired visual guidance of reach within his sighted upper visual field was evident.
He naturally compensates for this by reaching along surfaces, such as his table, or the floor, as this limits the task of reaching to two dimensions and not three. The floor or the table provide supplementary tactile guidance.

His difficulty giving attention to more than one thing at once, is probably responsible for colliding with people and objects when he is distracted or tired. Going out together when it is quiet, exploring open countryside, not talking when walking are all ways to avoid collisions.

Accessing information
T1 is limited, by his low level of visual acuity of around 6/200 (see below), his lack of lower visual fields, and his probable inability to see more than one or two things at once.

The low visual acuity and contrast sensitivity, mean that up to a distance of 50cm at least only high contrast image material in which every meaningful element (such as the eyes and other facial features on toys or in pictures) can be seen, unless then are wider than 5mm and separated by more than 5mm.

This situation is compounded by the concept that only single parts of pictures may be seen. It is worth assuming that the picture of the face has to be built up bit by bit during close viewing. This

may well explain why T1 can hold someone's face in his hands to stabilise the image, and thus to sequentially build up the whole picture in his mind. This is however a hypothesis only.

It is clear from this account that T1 is potentially a sequential learner, as he builds up simple clear meaningful information in his own mind.

Delivery of education needs to take all these issues into account by ensuring:

- That only salient items are present in his clutter and pattern free learning environment
- That T1 is taught by ensuring that all material used are visible by being large enough, of high contrast, in the sighted visual field.
- That the approach used is sequential and aimed at enhancing each experience immediately after it has taken place, with a pace matched to his processing speed (see below)

In this way T1 is likely to progressively learn.
Even when T1 is apparently amusing himself, it is worth remembering his presence, and regularly give him special attention in such a way that all members of the family at home, or staff at school communicate as 'radio communicators' at a speed matched to that which causes him to still and apparently focus. This is most likely to prove effective when he is not being distracted by anything else.

Processing of sound

While this account primarily considers T1's vision, it is essential recognise that sight sound smell and touch are all integrated into a meaningful whole. (Whether it is the appearance, the feel, or the sound of a drum, each element contributes to its identification and knowledge of its purpose.)

It is in this context that it is relevant to include mention of how sound information is likely to be processed in different ways to what is typical in this author's experience.

1. The majority of children that show T1's clinical picture of vision, are unable to accurately determine where sound is coming from.
2. Many such children who have not developed language respond by completely stilling when the elements of speech are deliberately prolonged. T1 is no exception. When speech was slowed to approximately half pace in all its elements, T1 became completely still, was no longer distracted and appeared to be listening intently, but when the speech was speeded up again, he once more became distractible.
(Such an observation is common in the children who, like T1, do not appear use vision to follow or respond to fast moving targets. This implies that T1 processes information, whether it is sight or sound, more slowly than others, and information that is presented more quickly than his processing speed is 'not there' and therefore inaccessible and not learnable from.)

The relevance of these observations is self-explicit, and is mentioned above.

Conclusion

T1's visual limitations significantly impact on T1's potential quality of life and his learning. They must of course be interpreted and acted upon in the context of his intellect and his ability to interpret his auditory environment.

Parenting and educational approaches that recognise T1's extant limitations are likely to lead to learning that is significant and rewarding

Appendix

Methods used and results obtained

Test for spectacles
T1 shows a small degree of long sightedness in each eye, but not enough to warrant spectacle prescription at this juncture

Visual acuities
T1's binocular visual acuity was found to be approximately 6/200 using both Cardiff cards* (at 25 cm) and Lea Gratings (at 25cm).

Contrast sensitivities
The binocular contrast sensitivity was found to be 46% using Cardiff contrast cards*

(*Both sets of Cardiff cards were held horizontally, and responses were sought and observed, owing to the lack of lower visual field, potentially abrogating meaningful responses being obtained.)

Visual fields
A 2cm and a 5cm worth ball were used to estimate the visual fields on the basis of when each ball was looked at.
Both balls were immediately seen in the upper visual field, but not in the lower visual field until they came up to the line of sight.

Visual guidance of movement of the lower limbs
T1 was observed as he walked around, and he collided with all low obstacles on the floor.

Visual guidance of movement of the upper limbs
T1 was observed as he reached out for items held in font of him, and he consistently reached short of them initially.

Age 9

Thanks for inviting me to reassess T1 and his vision today.

You had felt that his vision had improved significantly, both in terms of T1's ability to see smaller images and items, and with respect to his ability to perceive more within his visual scene and his ability to move through it.

The visual field is an assessment of the area over which one can see. As
expected T1 showed no evidence of seeing in his lower visual field below the horizontal midline, and when watching the computer screen he chose to get below it in order to ensure its maximum visibility. This compensation strategy is typical.

The visual acuity is a measure of how clearly one can see. T1 shows evidence of delayed visual maturation. From the measure of 6/200 when his acuity was last estimated the estimated measure today is 6/75.
This is consistent with the observation that he has become able to see fairly thin wisps of his teacher's hair, when initially he was unable to do so.

A number of aspects of behaviour and understanding were also apparent.
T1 was able to anticipate, to plan, to voice his needs, to show interest excitement and affection, and to purposefully manipulate the computer keyboard seeking out chosen elements of slowed YouTube favourite movies. These all represent significant developments.
