# Supplementary material for: The NR2F1-Related 5q14.3–q21.1 deletion causing periventricular heterotopia with cerebral visual impairment: a longitudinal case report and genotype–phenotype analysis
Source: Front Genet. 2026 May 7;17:1793726. doi: 10.3389/fgene.2026.1793726 (PMC13189929; doi:10.3389/fgene.2026.1793726)
Supplement: Supplementary file 4 [file DataSheet1.pdf]

Gene Symbol	OMIM Morbid Y/N	ClinGen AR/AD	T1 Relevance (Primary / Possible modifier / Unlikely / Unknown)
**ADGRV1**	Y	AR	Unlikely
**ARB2A**	N	None established	Unknown
**ARRDC3**	N	None established	Unknown
**ARSK**	Y	None established	Unlikely
**CETN3**	N	None established	Unknown
**CHD1**	Y	AD	Possible
**ELL2**	N	None established	Unknown
**ERAP1**	N	None established	Unknown
**ERAP2**	N	None established	Unknown
**FAM174A**	N	None established	Unknown
**FAM81B**	N	None established	Unknown
**GIN1**	N	None established	Unknown
**GLRX**	N	None established	Unknown
**GPR150**	N	None established	Unknown
**KIAA0825**	Y	None established	Unlikely
**LIX1**	N	None established	Unknown
**LNPEP**	N	None established	Unknown
**LYSMD3**	N	None established	Unknown
**MACIR**	N	None established	Unknown
**MBLAC2**	N	None established	Unknown
**MCTP1**	N	None established	Unknown
**NR2F1**	Y	AD	Primary
**NUDT12**	N	None established	Unknown
**PAM**	N	None established	Unknown
**PCSK1**	Y	None established	Unlikely
**POLR3G**	N	None established	Unknown
**POU5F2**	N	None established	Unknown
**PPIP5K2**	Y	AR	Unlikely
**RFESD**	N	None established	Unknown
**RGMB**	N	None established	Unknown
**RHOBTB3**	N	None established	Unknown
**RIOK2**	N	None established	Unknown
**SKIC3**	Y	AR	Unlikely
**SLCO4C1**	N	None established	Unknown
**SLCO6A1**	N	None established	Unknown
**SLF1**	N	None established	Unknown
**SPATA9**	N	None established	Unknown
**ST8SIA4**	N	None established	Unknown

This table lists all protein-coding genes located within the GRCh38-defined deletion interval in T1 (chr5:90,079,852–103,658,165). Gene–disease validity, inheritance pattern, and dosage sensitivity classifications were reviewed using OMIM and ClinGen at the time of manuscript preparation. Genomic interval and gene content were confirmed using the DECIPHER database (Foreman et al., 2023).

Primary: Gene with established monoallelic dosage sensitivity relevant to a heterozygous deletion and strong phenotypic concordance with T1.
Possible modifier: Gene with plausible neurodevelopmental relevance under autosomal dominant inheritance, but without established evidence as the primary driver of the observed phenotype in a contiguous gene deletion.
Unlikely: Genes associated primarily with autosomal recessive disorders or without established evidence of haploinsufficiency; not expected to contribute to phenotype in a heterozygous deletion context.
Unknown: Genes without established monogenic disease association or dosage sensitivity evidence.
